# Dr. McLaughlin’s Book

**Published:** 1893-06

**Authors:** 


DR. McLAUGHLIN'S BOOK.—"Fermentation Infection and Immunity" is, we are gratified to see, attracting much attention in Europe, and has received the compliment of a neat review at the hands of the two leading English medical periodicals, the *Journal* of the *British Medical Association* and the *London Lancet.* Doubtless it was somewhat of a surprise to our cousins across the water to see published in Texas the results of so extensive original research upon such a subject, and that too by an *amateur*, the subject being one that has heretofore engaged the attention only of bacteriologists among the *savants* of the old world. The work reflects credit upon its talented author; and the JOURNAL is pleased to see that though tardy, recognition of his claims as an original thinker has come at last, and Dr. McLaughlin is accorded position with the foremost investigators. As a matter of interest to our readers, and also from a feeling of State pride, we reproduce in this issue one of the notices referred to, that from the *London Lancet* (of April, 1892), and will give that of the *Journal* of the *British Medical Association* in our July number.

